# Reduced murine double minute-2 methylation from peripheral blood mononuclear cells correlates with enhanced oxidative stress in hepatitis b virus-related hepatocellular carcinoma

**DOI:** 10.3389/fmicb.2025.1590492

**Published:** 2025-05-13

**Authors:** Jing-Wen Wang, Han-Xu Zhu, Feng Zhang, He Wang, Yu-Chen Fan, Li-Yan Han, Kai Wang

**Affiliations:** ^1^Department of Hepatology, Qilu Hospital of Shandong University, Jinan, China; ^2^Institute of Hepatology, Shandong University, Jinan, China

**Keywords:** HBV-related hepatocellular carcinoma, murine double minute-2, oxidative stress, DNA methylation, metabolomics

## Abstract

**Background:**

Hepatitis B virus-related hepatocarcinogenesis (HBV-related HCC) involves a variety of causes including oncogene hypomethylation, oxidative stress and HBV itself. Oxidative stress induces an alternation in the DNA methylation status. We aimed to study the relationship between oxidative stress and murine double minute-2 (MDM2) methylation status in HBV-related HCC patients and healthy controls (HCs).

**Methods:**

A total of 135 patients with HBV-related HCC and 26 healthy controls (HCs) were recruited. The MDM2 methylation status was detected by methylation-specific PCR. The expression of MDM2 mRNA was assessed using quantitative real-time PCR. The plasma malondialdehyde (MDA), superoxide dismutase (SOD), glutathione (GSH), nuclear factor erythroid 2-related factor 2 (NRF2), heme Oxygenase-1 (HO-1), and glutathione peroxidase 4 (GPX4) were measured by enzyme-linked immunosorbent assay (ELISA). Thirty-six patients with HBV-related HCC and 11 HCs were selected and the serum metabolism was analyzed by ultra-high-performance liquid chromatography-mass spectrometry (UHPLC-MS).

**Results:**

Compared with HCs, the MDM2 promoter methylation frequency was significantly decreased in HBV-related HCC. The MDA levels were increased, whereas the GSH, SOD, NRF2, HO-1, and GPX4 levels were decreased in the HBV-related HCC patients relative to HCs. There were 216 differential metabolites between HBV-related HCC and HCs in plasma, which belongs to amino acids, bile acids, fatty acids, phospholipids, and other compounds. The cysteine and methionine metabolism were the most significant metabolic pathways associated with differential metabolites between MDM2 methylated group and MDM2 unmethylated group in HBV-related HCC.

**Conclusion:**

Our results suggested that oxidative stress may cause MDM2 hypomethylation, in which cysteine and methionine pathway might play an important role in.

## 1 Introduction

Hepatocellular carcinoma (HCC) is most common primary liver cancer and the third leading cause of cancer deaths worldwide (Bertuccio et al., 2017). Especially in China, HCC induced by chronic hepatitis B virus (HBV) infection is more universal (Huang et al., 2018). The HBV infection, activation of oncogene, oxidative stress are all pathogenesis of HCC (Nishida and Kudo, 2013; Zhao L. et al., 2019).

DNA methylation is a major epigenetic alterations in human cancer, which contains hypermethylation and hypomethylation (Patchsung et al., 2012; Klein Hesselink et al., 2018). The status of DNA methylation relies on the normal expression of DNA methyltransferases (DNMTs), DNA demethylases, histone modification enzymes, one-carbon metabolism, DNA integrity and cell proliferation (Valinluck and Sowers, 2007; Arita et al., 2008; Ooi and Bestor, 2008). Any of the factors affect the status of DNA methylation. DNA hypomethylation may lead to oncogene instability, promote increased expression of oncogene and lead to development of cancer (Aporntewan et al., 2011; Linabery et al., 2012).

Murine double minute-2 (MDM2) plays an important role in controlling transcriptional activity and protein stability through the MDM2-p53 pathway. Under normal conditions, MDM2 and p53 form an auto-regulatory feedback loop, which could keep a balance between them (Meng et al., 2014). Alterations in MDM2-p53 pathway are common in different tumors. Especially MDM2, as an oncogene, is overexpression in various cancers (Oliner et al., 2016; Muhseen and Li, 2019). In our previous study, we have found MDM2 hypomethylation in peripheral blood mononuclear cells (PBMCs) in hepatitis B virus-related HCC (HBV-related HCC) (Wang et al., 2020). However, the mechanism of MDM2 promoter hypomethylation remains unclear. Oxidative stress is an imbalance between oxidants and antioxidants and is the cause of numerous cancers (Klaunig, 2018). Oxidative stress not only plays a key role in hepatocarcinogenesis but also leads to epigenetic alteration. Several studies have shown that oxidative stress is associated with DNA hypomethylation, such as bladder and prostate cancer (Bhusari et al., 2010; Kloypan et al., 2015). DNA hypomethylation and oxidative stress are coupled via one-carbon metabolism and transsulfuration pathway. Oxidative stress leads to GSH depletion, which cannot provide sufficient methyl groups for DNA (Menezo et al., 2016; Sobočan et al., 2019). Oxidative stress can be measure by detecting the levels of MDA, the antioxidant enzymes and the differential metabolites. Metabolomics is a study of endogenous metabolites of small molecular mass, downstream of genomics, transcriptomics and proteomics. Changes in the metabolomics not only reflect the changes of genomics and proteomics, but also are affected by environmental factors, which reflects more comprehensively (Lindon and Nicholson, 2008; Patti et al., 2012; Hashim et al., 2019). The untargeted metabolomics by liquid chromatograph mass spectrometer (LC-MS) has demonstrated significance differences in bile acids, phospholipids, fatty acids, glycolysis, and urea cycles in serum, plasma, urine, and fecal samples from HCC patients compared with healthy controls (HCs) (Patterson et al., 2011; Ressom et al., 2012; Xiao et al., 2012). Oxidative stress can change the kinds and content levels of metabolites, and metabolites can cause changes in DNA methylation status (Andrisic et al., 2018; Zaghlool et al., 2020). We have pointed out that MDM2 hypomethylation plays an important role in HBV-related HCC occurrence. Even though MDM2 methylation status and increased oxidative stress are all play a role in diseases, no relationship has yet been explored between them.

In this study, we detected the methylation status of MDM2 in the PBMCs from the HBV-related HCC patients and the HCs. We determined the oxidative stress parameter levels in plasma of the patients with HBV-related HCC and HCs. At the same time, differences in metabolites between the two groups were compared. We aimed to identify the association between MDM2 methylation status and oxidative stress in HBV-related HCC.

## 2 Materials and methods

### 2.1 Patients

We enrolled in 135 patients with HBV-related HCC from the Department of Hepatology, Qilu Hospital of Shandong University, between June 2016 to August 2019. In addition, we recruited 26 HCs. The HBV-related HCC patients fulfilled the 2010 update of the American Association for the Study of Liver Diseases Practice Guidelines for Management of hepatocellular carcinoma (Bruix and Sherman, 2011). The HCs without prior history of hepatitis B infection and negative for HBV antibodies.

Informed consent was obtained from all individual participants included in the study. All procedures of this study were in accordance with the Declaration of Helsinki. Approval for the study was obtained from the Medical Ethics Committee of Shandong University Qilu Hospital.

### 2.2 Clinical parameters

HBV DNA load, hepatitis e surface antigen (HBe Ag), alanine aminotransferase (ALT), aspartate aminotransferase (AST), total bilirubin (TBIL), albumin (ALB), and prothrombin time (PT) were conducted in the Department of Laboratory Medicine, Qilu Hospital, Shandong University.

### 2.3 Separation of plasma and PBMCs

Five milliliters of venous peripheral blood were collected from each participant. Plasma was obtained following centrifugation of venous peripheral blood, and stored at −80°C until measurement. PBMCs were isolated with Ficoll-Paque (Pharmacia Diagnostics, Uppsala, Sweden) following the instruction.

### 2.4 DNA and RNA extraction

Genomic DNA was extracted from PBMCs using QIAamp DNA Mini Kit (Qiagen, Hilden, Germany). Total RNA was extracted by phenol-chloroform-isopropanol method. RNA was reverse transcribed into cDNA using the First-Strand cDNA Synthesis Kit (Fermentas, Vilnius, Lithuania). DNA and cDNA were stored in a −80°C refrigerator until used.

### 2.5 Sodium bisulfite modification and methylation-specific polymerase chain reaction

DNA bisulfite modification was performed by the EZ DNA Methylation-Gold kit (Zymo Research, Irvine, CA, United States), and finally 20 μL modified DNA was obtained for MSP or stored −80°C for standby application.

The MDM2 specific primers were designed by MethPrimer 2.0. MSP primer sequences are shown in Table 1. The total volume was 25 μL, which contains 1 μL modified DNA, 0.5 μL forward and reverse primers (10 μM), 12.5 μL Premix Taq (Zymo Research, CA, United States), and 10.5 μL nuclease-free water. The PCR program included initial denaturation at 95°C for 5 min, followed by 35 cycles of 95°C for the 30 s, 58.5°C for 30 s, and 72°C for 30 s; and a final extension at 72°C for 10 min. The negative control was nuclease-free water without DNA. Ten microliter PCR products were isolated on a 2% agarose gel, stained with Gel Red (Biotium, California, United States), and the electrophoresis results were visualized under UV illumination.

**TABLE 1:** MSP primer sequences of MDM2 gene.

Primer name	Primer sequence (5′–3′)	Annealing temperature (°C)	Product size (bp)
M	F: TAACGGTTAAAGG AGTGTTATAGCG R: GAAATAAAAAT ATTAACCGCGAAC	58.5	93
U	F: AATGGTTAAAGGA GTGTTATAGT R: CAAAATAAAAA TATTAACCACAAAC	58.5	93

### 2.6 Quantitative real-time polymerase chain reaction

The total volume of 20 μL containing 10 μL SYBR Green premix, 8.2 μL nuclease-free water, 1 μL cDNA and 0.4 μM of forward and reverse primers. The qRT-PCR protocol was composed of denaturation at 95°C for the 30 s, followed by 40 cycles of 95°C for 5 s, 60°C for 30 s, and 72°C for 30 s. The qRT-PCR primers were as follows: MDM2: forward primer 5′-GGGAGTGATCAAAAGGAC-3′ and reverse primer 5′-CCAAATGTGAAGATGAAGGTTTC-3′ (Pasello et al., 2015), β-actin: forward primer 5′-ATGGGTCAGAAGGATTCCTATGTG-3′, and reverse primer 5′-CTTCATGAGGTAGTCAGTCAGGTC-3′. β-actin was used as an internal reference gene.

### 2.7 Enzyme-linked immunosorbent assay

In this study, the levels of plasma malondialdehyde (MDA), superoxide dismutase (SOD) and glutathione (GSH) were detected. The following kits were used for quantitative determination of oxidative stress parameters in plasma: MDA ELISA Kit (Shanghai Lengton Bioscience Co., Ltd., China), SOD ELISA Kit (Shanghai Lengton Bioscience Co., Ltd., China), GSH ELISA Kit (Shanghai Lengton Bioscience Co., Ltd., China), NRF2 ELISA Kit (Shanghai Lengton Bioscience Co., Ltd., China), HO-1 ELISA Kit (Shanghai Lengton Bioscience Co., Ltd., China), GPX4 ELISA Kit (Shanghai Lengton Bioscience Co., Ltd., China). The specific operation methods were performed according to the manufacturer’s instruction.

### 2.8 Ultra-high-performance liquid chromatography-massspectrometry

Thirty-six patients with HBV-related HCC and 11 HCs were selected for detection of metabolites in plasma by the ultra-high-performance liquid chromatography-mass spectrometry (UHPLC-MS). Fifty microliter of plasma and 200 μL of extract solution (acetonitrile: methanol = 1:1, containing isotopically-labeled internal standard mixture) were mixed, followed by vortexed for 30 s, sonicated for 10 min in ice-water bath, and incubated for 1 h at −*40*°C to precipitate proteins. The supernatant was obtained by centrifugation (4°C, 12,000 r/min, 15 min). Six quality control (QC) sample were obtained by mixing the supernatant of each sample. The LC/MS-MS analysis was detected by UHPLC system (Vanquish, Thermo Fisher Scientific) with a Waters ACOUITY UPLC Amide column coupled to the Exactive HFX mass spectrometer (Orbitrap MS, Thermo). The injection volume was 3 μL.

### 2.9 Statistical analysis

The continuous variables were expressed as median (centile 25; centile 75). The differences between continuous variables were analyzed by Mann-Whitney U-test. Categorical values were presented by relative frequencies. The Chi-square test was applied to categorical data. The correlation between difference variables were analyzed by the Spearman correlation. The two-sided *p* < 0.05 was considered statistically significant. Statistical analysis was performed using SPSS version 19.0 software (SPSS, Chicago, IL, United States) and GraphPad Prism 6.0 (San Diego, CA, United States).

The raw data were converted to the mzXML format using ProteoWizard and processed by R package XCMS. Data obtained from metabolomics were imported into the SIMCA software (V16.0.2, Umea, Sweden) for principal component analysis (PCA), orthogonal partial least squares discrimination analysis (OPLS-DA). The quality of the PCA and OPLS-DA models was evaluated based on R^2^Y and *Q*^2^-values (R^2^Y: interpretation rate of the model, Q^2^: predictability of the model). Metabolites with *p* < 0.05, variable importance for the projection (VIP) > 1, fold change (FC) < 2 or < 0.5 were screened as significant different metabolites. The two-sided *p*< 0.05 was considered statistically significant.

## 3 Results

### 3.1 Patient characteristics

In this study, we recruited 135 patients with HBV-related HCC and 26 HCs. Nineteen patients with HBV-related HCC were excluded for co-infection with other diseases (9), existing of other tumors (3), incomplete data (7). The baseline participants characteristics are shown in Table 2.

**TABLE 2:** The baseline characteristics of the subjects.

Variable	HBV-related HCC (*n* = 135)	HCs (*n* = 26)	*p*-value
Gender (M/F)	111/24	14/12	0.001
Age (years)	59 (52–64)	28 (25.75–30.25)	<0.001
Log_10_[HBV DNA]	3.67 (2.98–5.15)	NA	NA
HBe Ag (+/−)	57/78	NA	NA
ALT (U/I)	42 (29–65)	27.5 (22.75–32.25)	<0.001
AST (U/I)	51 (39–94)	25 (20.00–28.00)	<0.001
TBIL (μmol/L)	20.5 (14.1–34.2)	14(8.75–17.83)	<0.001
ALB (g/L)	37.1 (32.9–41.1)	44.55(40.98–48.15)	<0.001
PT (s)	13 (12.2–14.5)	11.6(10.48–12.60)	<0.001
MDM2 methylation	41 (30.37%)	23 (88.46%)	<0.001

Quantitative variables are expressed as the median (centile 25; centile 75). HBV-related HCC, hepatitis B virus-related hepatocellular carcinoma; HCs, healthy controls; HBe Ag, hepatitis e surface antigen; ALT, alanine aminotransferase; AST, aspartate aminotransferase; TBIL, total bilirubin; ALB, albumin; PT, prothrombin time; MDM2, murine double minute-2; NA, not available.

### 3.2 Methylation frequency of MDM2 in the HBV-related HCC patients and HCs

**FIGURE 1:**
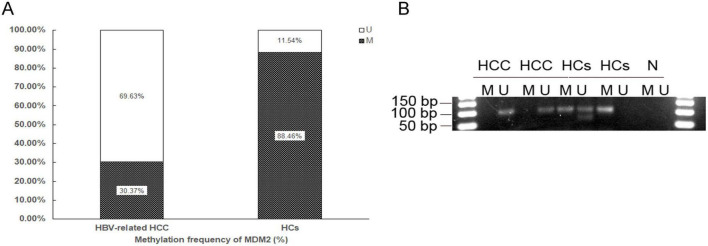
Aberrant methylation of MDM2 gene promoter in patients. Methylation status of MDM2 in the HBV-related HCC and HCs **(A)** and agarose gel electrophoresis results of MSP products of MDM2 **(B)**. HBV-related HCC, hepatitis B virus-related hepatocellular carcinoma; HCs, healthy controls; NC negative control, M methylated sequence; U, unmethylated sequence.

We detected the MDM2 methylation status in the HBV-related HCC patients and HCs. The methylation frequency was significantly decreased in the HBV-related HCC patients compared with HCs (30.37 vs. 88.46%, *p* < 0.001) (Figure 1A). The results of MSP are shown in Figure 1B.

Next, we analyzed the relationship between MDM2 methylation in PBMCs and clinical features from the patients with HBV-related HCC. We observed that the MDM2 methylation was associated with gender (*p* = 0.021), AFP (*p* = 0.011), number of tumors (*p* = 0.002), tumor size (*p* = 0.001), vascular invasion (*p* = 0.040) and TNM stage (*p* = 0.037) (Table 3). However, there was no significantly difference in age.

**TABLE 3:** Relationship between the MDM2 methylation and clinical features in the HBV-related HCC patients.

Variable	Number	Methylated	Unmethylated	χ^2^	*p*-value
Gender				5.319	0.021^a^
Male	111	29 (26.13%)	82 (73.87%)		
Female	24	12 (50%)	12 (50%)		
Age				0.121	0.728^a^
≥ 50	111	33 (29.73%)	78 (70.27%)		
< 50	24	8 (33.33%)	16 (66.67%)		
AFP (ng/mL)				6.517	0.011^a^
≥ 20	75	16 (21.33%)	59 (78.67%)		
< 20	60	25 (41.67%)	35 (58.33%)		
Number of tumors				9.765	0.002^a^
Multiple	67	12 (17.91%)	55 (82.09%)		
Solitary	68	29 (42.65%)	39 (57.35%)		
Tumor size (cm)				11.154	0.001^a^
> 5	40	4 (10.00%)	36 (90.00%)		
≤ 5	95	37 (38.95%)	58 (61.05%)		
Vascular invasion				4.205	0.040^a^
Yes	36	8 (22.22%)	28 (77.78%)		
No	99	41 (41.41%)	58 (58.59%)		
TNM stage				4.357	0.037**^a^**
I + II	64	25 (39.06%)	39 (60.94%)		
III + IV	71	16 (22.54%)	55 (77.46%)		

AFP, alpha fetoprotein; TNM, tumor node metastasis.

^a^Chi-square test.

### 3.3 MDM2 mRNA levels in the HBV-related HCC patients and HCs

**FIGURE 2:**
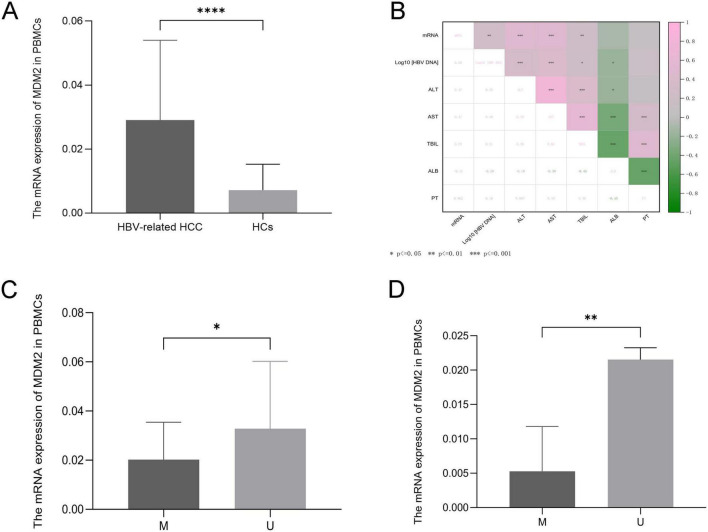
Relative expression levels of MDM2 in the HBV-related HCC and HCs and their correlation with clinicopathological parameters. Expression levels of MDM2 in the HBV-related HCC and HCs **(A)**. MDM2 mRNA levels were positively correlated with HBV DNA, ALT, AST and TBIL **(B)**. Expression of MDM2 mRNA in the methylated group was significantly lower than that in the unmethylated group both in the HBV-related HCC and HCs **(C,D)**. *p < 0.05, **p < 0.01, ***p < 0.001, ****p < 0.0001.

We found that the mRNA levels of MDM2 were increased in the HBV-related HCC patients compared with HCs (*p* < 0.0001) (Figure 2A). Next, we assessed the relationship between MDM2 mRNA levels and clinical features in HBV-related HCC patients. The MDM2 mRNA levels showed a significant positive correlation with HBV DNA (*p* = 0.002), ALT (*p* < 0.001), AST (*p* < 0.001), and TBIL (*p* = 0.009) respectively, and no obvious correlation between MDM2 mRNA levels and ALB (*p* = 0.185) or PT (*p* = 0.472) (Figure 2B and Table 4).

**TABLE 4:** Relationship between the MDM2 mRNA levels and clinical features in the HBV-related HCC patients.

Clinical features	mRNA	Log_10_ [HBV DNA]	ALT	AST	TBIL	ALB	PT
mRNA	1.000						
Log_10_[HBV DNA]	0.264**	1.000					
ALT	0.471**	0.348**	1.000				
AST	0.468**	0.397**	0.794**	1.000			
TBIL	0.224**	0.210*	0.385**	0.614**	1.000		
ALB	−0.115	−0.200*	−0.190*	−0.378**	−0.450**	1.000	
PT	0.062	0.161	0.067	0.303**	0.480**	−0.452**	1.000

*p < 0.05,

** *p* < 0.01, *** *p* < 0.001.

To clarify the effect of methylation on transcription, we explored the relationship between MDM2 methylation and MDM2 mRNA levels in subgroup. We observed that the mRNA levels of MDM2 were significantly decreased in the methylated group compared to the unmethylated group in the HBV-related HCC patients and HCs (HBV-related HCC: *p* = 0.047; HCs: *p* = 0.005) (Figures 2C,D).

### 3.4 Plasma oxidative stress parameters in the HBV-related HCC patients and HCs

**FIGURE 3:**
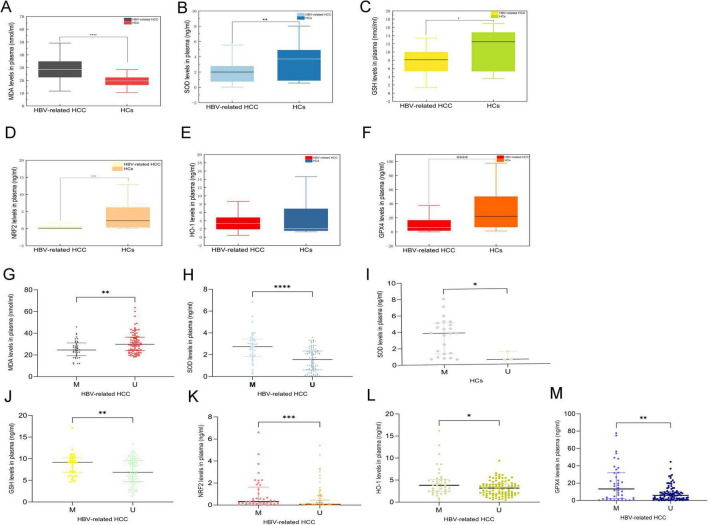
Oxidative and antioxidative parameters in the HBV-related HCC and HCs. MDA levels of patients with HBV-related HCC were significantly increased compared with HCs **(A)**. Antioxidative stress parameters were lower in HBV-related HCC than that in HCs **(B–F)**. MDA levels in the MDM2 methylated group with the HBV-related HCC were decreased compared with the MDM2 unmethylated group **(G)**. However, antioxidative stress parameters levels in the MDM2 methylated group with the HBV-related HCC were increased compared with the MDM2 unmethylated group **(H,J–M)**. SOD levels in the MDM2 methylated group were higher than those in the unmethylated group in the HCs **(I)**. *p < 0.05, **p < 0.01, ***p < 0.001, ****p < 0.0001.

Then, we studied various parameters of oxidative stress in plasma. Our results showed that MDA levels were significantly increased in the HBV-related HCC patients compared to HCs (MDA: *p* < 0.0001) (Figure 3A). Furthermore, the levels of antioxidants SOD, GSH, NRF2, HO-1, and GPX4 in plasma were lower in the HBV-related HCC patients than that in the HCs, particularly with significant reductions in SOD, GSH, NRF2, and GPX4 (SOD: *p* = 0.005; GSH: *p* = 0.021; NRF2: *p* < 0.0001; HO-1: *p* = 0.4405; GPX4: *p* < 0.0001) (Figures 3B–F).

### 3.5 Correlation of MDM2 methylation status with oxidative stress

To determine whether there was a relationship between oxidative stress and the MDM2 methylation in the HBV-related HCC patients and HCs, we next evaluated the correlation between the MDM2 methylation and oxidative stress levels in plasma. We found that the plasma MDA levels were significantly higher in MDM2 unmethylated group than in MDM2 methylated group in the HBV-related HCC patients (MDA: *p* = 0.001) (Figure 3G). Moreover, the levels of SOD, GSH, NRF2, HO-1, and GPX4 were decreased in MDM2 unmethylated group compared with MDM2 methylated group in the plasma of the HBV-related HCC patients (SOD: *p <* 0.0001; GSH: *p* = 0.003; NRF2: *p* = 0.0002; HO-1: *p* = 0.017; GPX4: *p* = 0.006) (Figures 3H,J–M). It is interesting that SOD, which was increased in MDM2 methylated group than in MDM2 unmethylated group in HCs (*p* = 0.024) (Figure 3I). However, there was no relationship between MDA, GSH, NRF2, HO-1, and GPX4 and MDM2 methylation status in HCs (*p* > 0.05).

### 3.6 Plasma metabolomic changes in the HBV-related HCC patients and HCs

**FIGURE 4:**
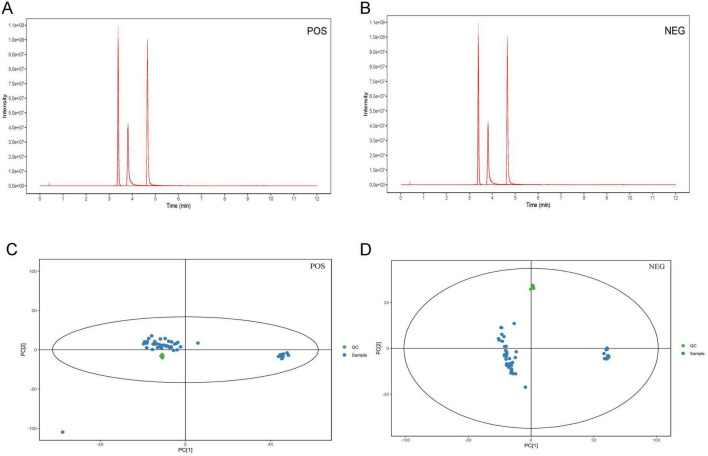
TIC and PCA of the quality control. The TIC in the positive **(A)** and negative ion model **(B)**. The PCA in the positive **(C)** and negative ion model **(D)**. TIC, total ion chromatography; PCA, principal component analysis; POS, positive ion model; NEG, negative ion model.

The results of total ion chromatography (TIC) and PCA of the QC were shown in Figure 4, which suggested that the instruments were stable and the experimental data were reliable for next analysis.

**FIGURE 5:**
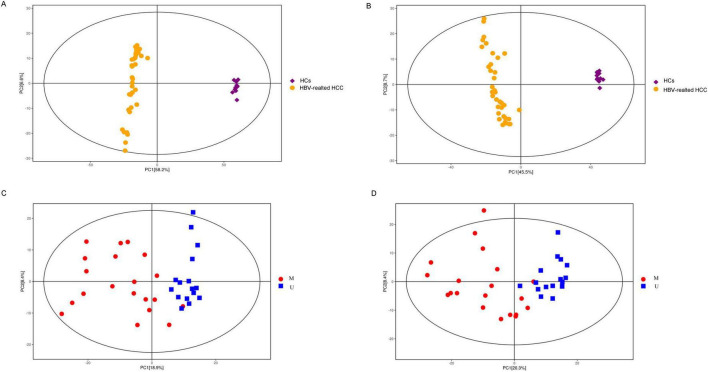
PCA score plots of the tested samples. PCA score plots for positive **(A)** and negative ion model **(B)** of HBV-related HCC and HCs. PCA score plots for positive **(C)** and negative ion model **(D)** of the MDM2 methylated group and MDM2 unmethylated group in HBV-related HCC. PCA, principal component analysis.

**FIGURE 6:**
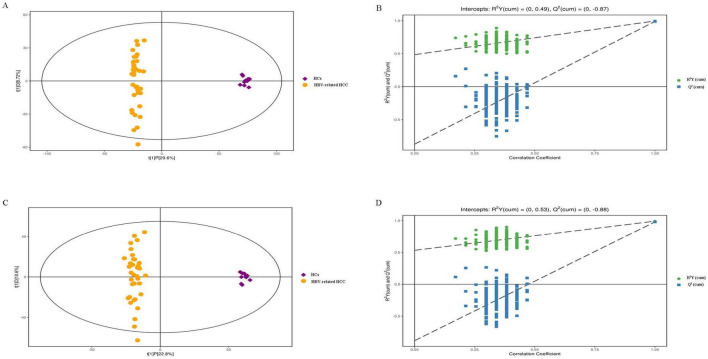
OPLS-DA analysis of plasma samples from HBV-related HCC and HCs. OPLS-DA score chart and permutation test in positive **(A,B)** and negative ion model **(C,D)**. OPLS-DA, orthogonal partial least squares discrimination analysis; R2Y, interpretation rate of the model, Q2, predictability of the model.

**FIGURE 7:**
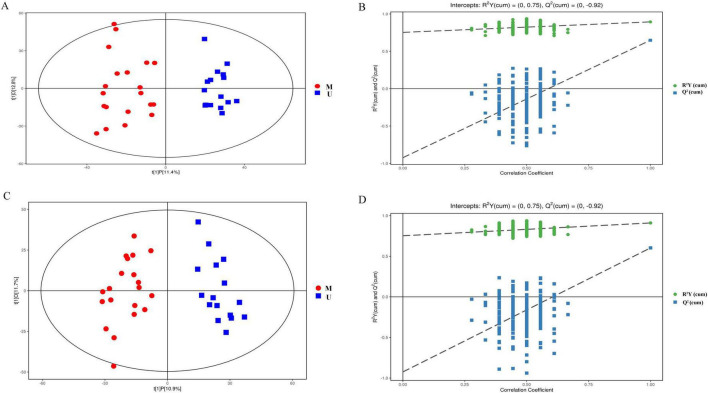
OPLS-DA analysis of plasma samples from the MDM2 methylated group and MDM2 unmethylated group in HBV-related HCC. OPLS-DA score chart and permutation test in positive **(A,B)** and negative ion model **(C,D)**.

**FIGURE 8:**
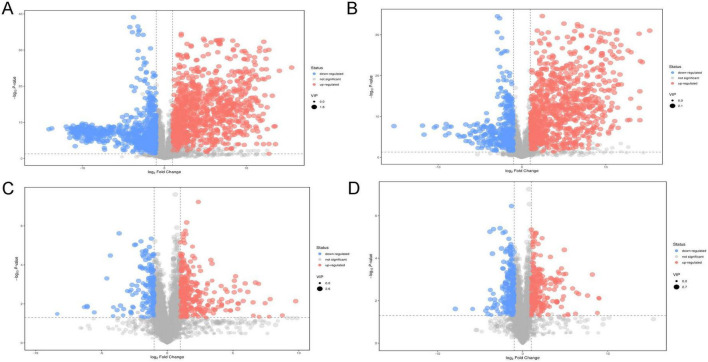
Volcanic map of differential metabolites in plasma of the tested samples. Volcano plots of differential metabolites in positive **(A)** and negative ion models **(B)** of HBV-related HCC and HCs. Volcano plots of differential metabolites in positive **(C)** and negative ion models **(D)** of the MDM2 methylated group and MDM2 unmethylated group in HBV-related HCC.

The PCA analysis between plasma in the HBV-related HCC group and HCs group showed obvious trend of separation, indicating significant metabolic differences between the two groups (Figures 5A,B). There was also a trend of separation between MDM2 methylated group and MDM2 unmethylated group in HBV-related HCC (Figures 5C,D). Next, the OPLS-DA analysis was established to refine the separation results obtained by PCA. It showed that the two group were obvious difference, the sample was basically in the 95% confidence interval (95% CI) (Figures 6A,C, 7A,C). In positive ion model, R^2^Y-value was 0.996, *Q*^2^-value was 0.989 (Figure 6B); in negative ion model, R^2^Y-value was 0.992, *Q*^2^-value was 0.980 in HBV-related HCC group and HCs group (Figure 6D). In positive ion model, R^2^Y-value was 0.894, *Q*^2^-value was 0.648 (Figure 7B); in negative ion model, R^2^Y-value was 0.910, *Q*^2^-value was 0.603 in MDM2 methylated group and MDM2 unmethylated group of HBV-related HCC (Figure 7D). A total 216 differential metabolites, 144 in positive ion model and 72 in negative ion model, were identified in plasma between the HBV-related HCC and HCs. Based on *p-*value and FC value, volcanic maps were constructed (Figures 8A,B). In positive ion model, 88 differential metabolites were significantly increased and 56 differential metabolites were significantly decreased in plasma of HBV-related HCC patients. In negative ion model, 51 differential metabolites were significantly increased and 21 differential metabolites were significantly decreased in plasma of HBV-related HCC patients. The compounds identified belongs to amino acids, bile acids, fatty acids, phospholipids, organic acids, sugars, organic heterocyclic compounds and other compounds. Representative differential metabolites are presented in Table 5.

**TABLE 5:** The differential metabolites in plasma between HBV-related HCC and HCs.

Variable	Compounds	RT (min)	Exptl (m/z)	VIP value	*p*-value	Fold change	HCC vs. HCs
Organic heterocyclic molecule	Bilirubin	54.648	585.2698	1.800	<0.001	10714.636	↑
	Urobilin	296.414	591.317	1.480	0.049	6966.897	↑
Amino acid	D-Ornithine	526.970	133.097	1.638	<0.001	9.714	↑
	Citrulline	405.828	176.103	1.483	<0.001	3.489	↑
	γ-glutamylleucine	423.307	261.144	1.418	<0.001	2.627	↑
	DL-Homocystine	418.315	267.047	2.085	<0.001	418.315	↑
	D-Aspartic acid	424.562	132.030	2.021	<0.001	13.599	↑
Bile acid	Glycocholic acid	267.450	464.301	1.012	0.043	12.918	↑
Hospholipid	Phosphatidyl cholines (24:0/20:5(5Z,8Z,11Z,14Z,17Z))	151.362	892.674	1.034	<0.001	2.915	↑
	Phosphatidyl inositols (18:1(11Z)/16:1(9Z))	213.868	835.531	1.394	<0.001	2.641	↑
	Phosphatidyl ethanolamines (16:0/18:2(9Z,12Z))	38.4037	716.520	1.136	<0.001	2.377	↑
Amino acid	Creatine	365.858	132.077	1.276	<0.001	0.311	↓
Phospholipid	Glycerol hosphocholine	403.837	258.109	1.128	0.001	0.463	↓
Organic acid	Malonic acid	103.003	90.598	1.426	<0.001	0.229	↓
Vitamin	Ascorbic acid	175.024	87.277	1.010	<0.001	0.296	↓

VIP, variable importance for the projection.

We also detected the differential metabolites in plasma between MDM2 methylated group and MDM2 unmethylated group in HBV-related HCC patients, and constructed volcanic maps based on *p*-value and FC value (Figures 8C,D). In positive ion model, 33 differential metabolites were significantly increased and 19 differential metabolites were significantly decreased. In negative ion model, 29 differential metabolites were significantly increased and 19 differential metabolites were significantly decreased. The compounds identified belongs to amino acids, fatty acids, phospholipids, bile acids, organic acids, and other compounds. Representative differential metabolites are presented in Table 6.

**TABLE 6:** The differential metabolites in plasma between MDM2 methylated group and MDM2 unmethylated group.

Variable	Compounds	RT (min)	Exptl (m/z)	VIP value	*p*-value	Fold change	M vs. U
Fatty acid	Resolvin D1	185.140	359.220	2.220	0.007	873.061	↑
Amino acid	S-adenosylmethionine	489.588	399.143	1.082	0.029	4.275	↑
Alkaloid	Leucinic acid	115.947	131.071	1.361	<0.001	2.644	↑
Sugars	Urocanic acid	299.954	139.050	1.230	0.019	2.235814537	↑
Oganic acid	D-Glucuronic acid	387.926	193.035	1.725	0.014	4.686	↑
	Indoxyl sulfate	27.226	212.002	2.310	<0.001	0.357	↓
Other compounds	3,4-Dihydroxybenzaldehyde	35.930	137.024	1.464	0.027	0.325	↓
	p-Cresol sulfate	23.975	187.007	1.642	0.012	0.405	↓
	4-Hydroxyhippuric acid	250.125	194.046	1.618	0.008	0.489	↓

VIP, variable importance for the projection.

### 3.6 Related pathways of differential metabolites

**FIGURE 9:**
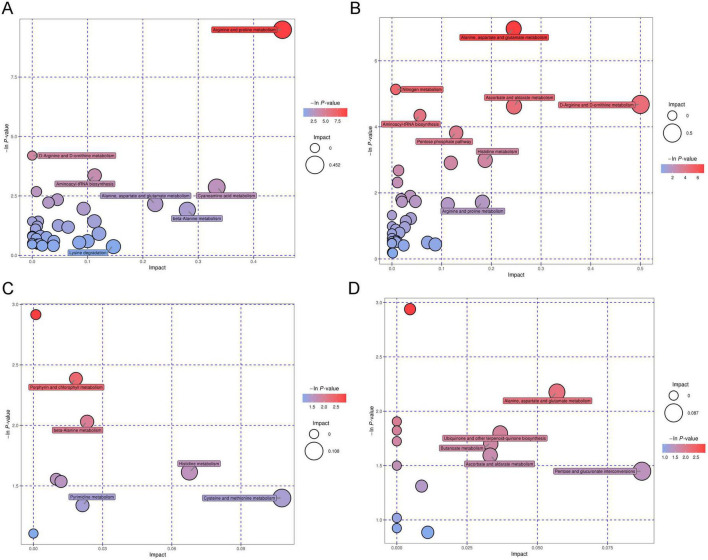
Metabolic pathway enrichment analysis of differential metabolites performed by KEGG of the tested samples. Metabolic pathway in positive **(A)** and negative ion models **(B)** of HBV-related HCC and HCs. Metabolic pathway in positive **(C)** and negative ion models **(D)** of the MDM2 methylated group and MDM2 unmethylated group in HBV-related HCC.

The pathway analysis of differential metabolites was performed using the Kyoto Encyclopedia of Genes and Genomes (KEGG) database. Among the HBV-related HCC and HCs, the differential metabolites were involved in the arginine and proline metabolism, D-Arginine and D-ornithine metabolism, alanine, aspartate and glutamate metabolism, nitrogen metabolism, and other pathways (Figures 9A,B).

The plasma metabolomics revealed differential metabolites were mainly involved in cysteine and methionine metabolism, histidine metabolism, pentose, and glucuronate interconversions, alanine, aspartate, glutamate metabolism and other pathways in MDM2 methylated group and MDM2 unmethylated group of HBV-related HCC (Figures 9C,D). The cysteine and methionine metabolism were the most significant metabolic pathways associated with differential metabolites between MDM2 methylated group and MDM2 unmethylated group in HBV-related HCC.

## 4 Discussion

In the present study, we showed that the methylation frequency of MDM2 in PBMCs was reduced in patients with HBV-related HCC compare with HCs. The plasma MDA levels in MDM2 unmethylated group were significantly higher than those in MDM2 methylated group, however, SOD, GSH, NRF2, HO-1, and GPX4 levels were significantly decreased in plasma of the unmethylated group compare with the methylated group in the HBV-related HCC patients. The differential metabolites related to antioxidant activity in MDM2 methylated group, such as resolvin D1 and S-adenosylmethionine, were higher than those in MDM2 unmethylated group. The differential metabolites associated with oxidant activity, such as indoxyl sulfate and p-cresol sulfate, were lower than those in MDM2 unmethylated group. In this study, we first demonstrated a potential correlation between the MDM2 hypomethylation and oxidative stress in the HBV-related HCC patients.

Hepatocarcinogenesis is considered a multistep process, in which chronic HBV infection, oxidative stress, and oncogene hypomethylation play a very essential role (Kim and Han, 2012; Zhao J. et al., 2019). PBMCs are composed of lymphocytes, monocytes and macrophages. Chronic inflammation is risk factor for HCC, in which lymphocytes play an important role in the development of chronic inflammation conditions (Yang et al., 2019). The recruitment of PBMCs is mainly due to the lymphocytes infiltration in the development of HCC. In addition, study has found that HBV can infect PBMCs *in vivo*, this result was also confirmed *in vitro* (Cabrerizo et al., 2000; Wang et al., 2018). Therefore, PBMCs are essential in cancer research. In this study, we found that the MDM2 methylation frequency was decreased compared with HCs as well as MDM2 mRNA levels were increased in PBMCs of HBV-related HCC patients. These results suggested that hypomethylation could result in the transcription of genes and the MDM2 is involved in the carcinogenesis of HBV-related HCC (Zhao J. et al., 2019). We also observed a correlation between the MDM2 methylation and the gender, AFP, number of tumors, tumor size, vascular invasion and TNM stage, which means MDM2 methylation might take part in the development of HBV-related HCC. Thus, these observations show a link between chronic HBV infection, MDM2 methylation and gene transcription in PBMCs of HBV-related HCC.

Chronic HBV infection not only causes the emergence of HCC, but also induces the oxidative stress in cells (Alavian and Showraki, 2016; Takeda et al., 2017). MDA, a marker for oxidative damage, has high cytotoxicity and inhibition of protective enzymes (Taysi et al., 2003). Besides, oxidative stress can lead to passive DNA demethylation, which results in epigenetic instability leading to tumors (Tsegay et al., 2019). Here, we found that MDM2 hypomethylation in the HBV-related HCC patients and the levels of MDA were significantly increased in the HBV-related HCC patients relative to HCs. In addition, MDA levels in MDM2 unmethylated group were higher than those in the MDM2 methylated group in HBV-related HCC patients. Taken together, these results provide evidence that increased MDA levels may play an important role in MDM2 hypomethylation in the HBV-related HCC.

On the other hand, decreased activity of antioxidant enzymes and a large amount of consumption GSH are also responsible for oxidative stress. SOD, GSH, NRF2, HO-1, and GPX4 are recognized as an effective markers for scavengers of oxidative stress (Himoto et al., 2010; Wang et al., 2025; Zhou et al., 2025). We found that the levels of SOD, NRF2, and GPX4 were significantly decreased and observed that GSH and HO-1 levels were also decreased in MDM2 unmethylated group of the HBV-related HCC patients. SOD, an essential enzyme that effectively scavenges oxygen free radicals, is related to the development and progression of cancer such as gastric cancer (Li et al., 2019). Under the normal conditions, S-adenosylmethionine (SAM) provides the homocysteine to synthesis of GSH and provides methyl group to maintain DNA methylation status. Under the oxidative stress, however, GSH synthesis requires a large amount of SAM resulting in insufficient SAM followed by DNA hypomethylation (Hitchler and Domann, 2007). Nrf2 is a key transcription factor in the cellular antioxidant stress response and can induce HO-1 and GPX4 (Deng et al., 2020; Zhou et al., 2025). NRF2 is a key transcription factor for maintaining oxidative homeostasis. It is activated under oxidative stress condition, promoting the transcription of target genes HO-1 and GPX4, while reducing ROS accumulation and DNA damage, which might be related to DNA methylation (Zhang et al., 2022).

Metabolomics is a comprehensive and systematic analysis of endogenous small molecule metabolites to demonstrate the overall effect of stimulation on the body. We compared the changes of plasma differential metabolites in HBV-related HCC and HCs, and found that the up-regulated differential metabolites in HBV-related HCC compared with HCs included bilirubin, urobilin and other heterocyclic compounds, D-ornithine, citrulline, γ-glutamylleucine, homocysteine and other amino acid, glycholic acid and other bile acids, phosphatidyl ethanolamine, phosphatidyl choline and phosphatidyl inositol and other lysophospholipids. Some studies indicate that excessive bilirubin can cause severe oxidative stress and tissue injury (Vodret et al., 2017). D-ornithine and citrulline may cause lipid peroxidation, and inhibit tricarboxylic acid cycle and oxidative phosphorylation (Camacho and Rioseco-Camacho, 1993; Viegas et al., 2011). Gamma- glutamylleucine is a marker of oxidative stress and reflective of GSH turnover (Metrustry et al., 2018). In our study, increased γ-glutamylleucine indicates intracellular oxidative stress and a large consumption of GSH in HBV-related HCC patients. Down-regulated metabolites were creatine, glycerol phosphocholine, malonic acid, ascorbic acid. Creatine can prevent oxidative stress and inflammation and could protect against tissue and mitochondrial DNA damage (Chilibeck et al., 2017). In our study, a decrease in plasma creatine maybe inhibitor the capacity of antioxidant and anti-inflammatory. In addition, the decreased of malonic acid of HBV-related HCC by inhibiting the malonic acid, thus affecting energy metabolism (Ahamad et al., 2017). These results indicated that HBV-related HCC exhibited specific metabolites changes and abnormal energy metabolism, tricarboxylic acid cycle, and oxidative phosphorylation.

We found significant differences in the levels of MDA, SOD, and GSH between the MDM2 methylated group and unmethylated group in HCC, so we compared the differential metabolites between the two group. The antioxidant metabolites resolvin D1, SAM, leucinic acid, urocanic acid, D-Glucuronic acid in MDM2 methylated group were significantly higher than those in unmethylated group. Some experiments showed that resolvin D1 has anti-inflammation and antioxidant effects, and increased the GSH levels, they can jointly play the role of antioxidative stress (Li et al., 2020). The levels of SAM were reduced in MDM2 unmethylated group of HCC, oxidative stress products increase GSH levels, which may influence DNA methylation by limiting the SAM availability leading to DNA hypomethylation (Oronsky et al., 2014; Thongsroy et al., 2017). We found that oxidative stress-related metabolites showed a decrease in the MDM2 unmethylated group of HCC, such as indoxyl sulfate, 3,4-dihydroxybenzaldehyde, p-cresol sulfate, 4-hydroxyhippuric acid. Indoxyl sulfate and p-cresol sulfate has been known to cause endothelial cell dysfunction by increasing ROS and oxidative stress (Sherzai and Elkind, 2015; Durgan et al., 2019). We unexpectedly found that 4-hydroxyhippuric acid in MDM2 methylated group were lower than that in MDM2 unmethylated group. 4-hydroxyhippuric acid is a microbial end product produced by polyphenol metabolism by intestinal microflora, and serum levels are affected by altered gut permeability, which indicated that the intestinal permeability may increase more significantly in MDM2 unmethylated group, but this needs to be further verified by subsequent experiments.

Together, we speculated that oxidative stress may be associated with MDM2 hypomethylation that promotes hepatocarcinogenesis. Several mechanisms might contribute to the MDM2 hypomethylation with oxidative stress: (1) Oxidative stress could change the activity and function of epigenetic related enzymes, such as DNA methyltransferases (DNMTs), to affect DNA methylation status. Kang et Al. demonstrated that oxidative stress may play a key role in gene promoter methylation, which may be associated with DNMT and histone deacetylase activity (Kang et al., 2012). (2) Due to the large amount of GSH synthesis, SAM is insufficient to maintain DNA methylation. (3) The cysteine and methionine metabolism might affect DNA methylation.

There are some limitations in this study. Firstly, we lack the MDM2 methylation status in liver tissues, as they are difficult to obtain. However, the study of PBMCs in HCC is also important. We will test the MDM2 promoter methylation in liver tissue samples in the future research. Secondly, we only demonstrated that MDM2 hypomethylation may be associated with oxidative stress. The exact mechanisms, however, need to be further verified *in vivo* and *vitro* experiments. Thirdly, HBV-related HCC is a major etiology of HCC but not the only cause of HCC, we will detect MDM2 methylation in HCC with other etiologies in subsequent experiments.

## 5 Conclusion

In conclusion, our study shows that the MDM2 hypomethylation of PBMCs might be associated with oxidative stress in HBV-related HCC patients. The cysteine and methionine pathway might play an important role in MDM2 hypomethylation. This study provides new insights into the epigenetic regulation induced by oxidative stress and may help to develop a new therapeutic strategy of the HBV-related HCC.

## Data Availability

The original contributions presented in the study are included in the article/supplementary material, further inquiries can be directed to the corresponding authors.
